# COVID Feel Good: Evaluation of a Self-Help Protocol to Overcome the Psychological Burden of the COVID-19 Pandemic in a German Sample

**DOI:** 10.3390/jcm11082080

**Published:** 2022-04-07

**Authors:** Marie Lisa Meyer, Arne Kaesler, Stefanie Wolffgramm, Nicolina Laura Perić, Gentian Bunjaku, Lilith Dickmann, Silvia Serino, Daniele Di Lernia, Cosimo Tuena, Luca Bernardelli, Elisa Pedroli, Brenda K. Wiederhold, Giuseppe Riva, Youssef Shiban

**Affiliations:** 1 Department of Psychology, Private University of Applied Science, 37073 Goettingen, Germany; meyer@pfh.de (M.L.M.); arne.kaesler@pfh.de (A.K.); stefanie.wolffgramm@yahoo.de (S.W.); peric@pfh.de (N.L.P.); gentian.bunjaku@gmx.de (G.B.); lilith.dickmann@web.de (L.D.); 2 Department of Psychology, Università Cattolica del Sacro Cuore, 20123 Milan, Italy; silvia.serino@gmail.com (S.S.); daniele.dilernia@unicatt.it (D.D.L.); 3 Humane Technology Lab, Università Cattolica del Sacro Cuore, 20123 Milan, Italy; cosimo.tuena@unicatt.it (C.T.); giuseppe.riva@unicatt.it (G.R.); 4 Applied Technology for Neuro-Psychology Lab, IRCCS Istituto Auxologico Italiano, 20149 Milan, Italy; e.pedroli@auxologico.it; 5 BECOME Srl, 20133 Milan, Italy; luca.bernardelli@become-hub.com; 6 Faculty of Psychology, University of eCampus, 22060 Novedrate, Italy; 7 Virtual Reality Medical Center, La Jolla, CA 92037, USA; b@vrphobia.eu; 8 Virtual Reality Medical Institute, 1200 Brussels, Belgium

**Keywords:** COVID-19, psychological burden, self-help, virtual reality, Germany

## Abstract

The COVID-19 pandemic has severe consequences for physical as well as mental well-being. In times of restricted social contact, online self-help programs offer a low-threshold first aid to cope with the psychological burden. This current study evaluates the online self-help protocol “COVID Feel Good” in a German sample. The multicentric study was designed as a single cohort with a waiting list control condition. The convenience sample consisted of 38 German individuals who experienced at least two months of restrictions during the COVID-19 pandemic. The 7-day self-help protocol included the VR video “Secret Garden” as well as a social or cognitive exercise each day. General distress, depression, anxiety, stress, and hopelessness were assessed as primary outcomes. Social connectedness and fear of coronavirus were measured as secondary outcomes. Results showed a significant decrease in all primary outcomes except for hopelessness. Furthermore, the results indicated a significant improvement in social connectedness. Treatment effects on general distress, depression, stress, and anxiety persisted for two weeks after participation. The present study indicates that VR-based self-help protocols can mitigate the psychological burden associated with the pandemic, supporting recent findings.

## 1. Introduction

Global pandemics take a toll on people’s physical as well as psychological well-being and are accompanied by different stressors, such as isolation, fear of infection, frustration, boredom, missing information, and insufficient supply [1].

In an attempt to contain the current COVID-19 pandemic, the German government implemented measures aimed at restricting social contacts and mandated an obligatory quarantine for anyone who tested positive for the disease as well as for their close contacts. With restricted personal movement and the shutdown of nearly all public life, people are confronted with an immense reduction in their personal relations. The restriction on social contacts bears the risk of increased social isolation and loneliness, which are in turn, linked to an increase in stress responses [2,3,4,5,6]. Therefore, it is assumed that prolonged periods of isolation and limited mobility due to the COVID-19 pandemic have an impact on mental well-being [7]. Negative emotional consequences include sadness, irritability, and mood swings [8]. Due to the additional restrictions imposed on the economic sector, many people also face stressful life events, such as unemployment [9] and financial difficulties, which can have an additional negative impact on mental health [10].

Recent studies conducted in Austria [11], Italy [12], Turkey [13], China [14,15], and Spain [16] pointed out a high prevalence of psychological burden during the COVID-19 pandemic. The results of similar studies conducted in Germany regarding anxiety, depression, and general distress are in line with these findings [17,18,19].

With no end in sight, the COVID-19 pandemic continues to put constant strain on people’s resources and coping capacities, making it an ongoing stressful experience [20]. Self-help programs can provide resources and strategies to better cope with crises such as the COVID-19 pandemic [21]. Taking into account the limited face-to-face support options in times of stringent lockdown measures, online mental health services are considered to be promising solutions in this regard [22] as they are easily accessible and can be broadly provided [23]. E-mental health interventions seem to be of great use for public education purposes regarding the potential influence of pandemic measures on psychological well-being [24]. They are already used successfully to reduce distress and to provide support in the times of the global COVID-19 pandemic [17,25].

Boosting individual resilience and self-efficacy is of key importance as they are negatively associated with adverse psychological outcomes during the COVID-19 pandemic [7,26]. To overcome the psychological burden of the COVID-19 pandemic, Riva et al. [8] developed an easy-to-use self-help protocol that is designed to help individuals to foster adaptive strategies at home. The aim of the self-help protocol *COVID Feel Good* is to generate relaxation and self-reflection through seven different daily exercises, one for each day of the program’s run. The basis of the 7-day-program is a ten-minute 360° virtual reality (VR) video displaying a simulated “*Secret Garden*”. It is designed to be a virtual retreat where emotional stress can be relieved. The narrative of the VR video aims to induce relaxation, based on the principles of compassion-focused therapy [27] which has in turn been proven to boost well-being and reduce depressive symptoms and stress by building up stress tolerance, empathy, and attention allocation [28,29,30]. Further, the self-help protocol *COVID Feel Good* is based on the empirical evidence that nature has positive effects on psychological well-being [31,32], especially for people suffering from depression or stress [33,34]. Nature is generally an easily accessible and freely available resource for most people. Due to the movement restrictions associated with the COVID-19 pandemic, accessibility to this resource is limited. However, simulated nature has the potential to influence mood and “replenish” depleted capacities [35] as well as induce positive emotions [36]. In the current study, the 360° video is supported by a head-mounted display to increase the immersiveness of the VR experience. High immersion creates a sense of presence which is a feeling of actually being inside the VR simulation and therefore seems to lead to greater therapeutic benefits [35]. Due to the high degree of immersion, the virtual world hardly presents any differences from the real world on the emotional and experiential level, which allows new possibilities for the strengthening of resources [35,37,38,39]. The daily social tasks of the *COVID Feel Good* protocol are inspired by Winch [40] and designed to support interpersonal relationships, personal resources, and coping strategies. Since these social tasks are practiced with a partner, they enable the experience of social connectedness and social support in dealing with negative emotions and situations, mitigating psychological burdens [41,42]; especially since social connectedness has proven to be a protective factor against stress, hopelessness, depressive symptoms, and worries concerning COVID-19 [1,41,43,44,45,46,47]. Moreover, the *COVID Feel Good* protocol aims at individuals’ mindfulness, which is in turn linked to a reduced hopelessness and fear of COVID-19 [48].

As part of a multi-center study, including samples from Italy, the USA, Spain, and Japan, the purpose of the current study was to scientifically evaluate the positive effect of the *COVID Feel Good* self-help protocol [8] on psychological well-being found by Riva et al. [49], in a German sample.

We expected the implementation of the VR self-help program to lead to a significant reduction in depression, anxiety, the fear of COVID-19, perceived stress, and hopelessness. The scores on the outcomes described should therefore be significantly lower after the intervention than before [8]. Treatment effects were also expected to be maintained at the two-week follow-up. Furthermore, we expected an increase in social connectedness as a result of the VR self-help program [8]. Finally, we expected a reduction in subjective distress as well as an increase in relaxation throughout the duration of the program [8].

## 2. Materials and Methods

### 2.1. Study Design

The current study was conducted in a within-subject pre-posttest design with a waiting list condition [49]. The primary and secondary outcomes (semi-trait measures) were measured one week before the start of the self-help protocol (day −7), on the day before the start of the protocol (day 0), at the end of the program (day 7), and at a two-week follow-up (day 21). Additionally, secondary outcomes (state measures) were assessed daily (day 1 to day 7) immediately after each exercise of the self-help program.

### 2.2. Participants

Participants were recruited via social media platforms, web presence, as well as newspaper and radio reports. In line with the pragmatic, real-life approach of the present study, a convenience sample was collected between January and May 2021. In contrast to the explanatory trials that focus on special, highly selected patient samples, the pragmatic trial *COVID Feel Good* is aimed at the general public, therefore broad eligibility criteria that reflect the heterogeneity of our target population were applied (see [8]). Participants had to meet the following inclusion criteria: age ≥18 years, German as a native language, access to a smartphone with a YouTube app, access to cardboard VR glasses, the commitment of a partner with whom to discuss the exercises (virtually or on-site), and experience with restriction measures or isolation of at least two months during the COVID-19 pandemic. The following exclusion criteria were set: uncorrected visual or hearing impairment and balance disorders. To ensure accessibility of the *COVID Feel Good* protocol for many individuals, people taking medication or suffering from other medical conditions were not excluded from study participation [8].

A sample size calculation was conducted using G*Power 3.1. Assuming an effect size of f = 0.25, an alpha significance level of α = 0.05, and a statistical power of 0.80, the required sample size was *N* = 36 [49].

For this trial, 40 participants were recruited, of which two dropped out before the start of the intervention phase. The final sample of *N* = 38 consisted of 10 males and 28 females with a mean age of *M* = 36.4 (*SD* = 12.5, min = 20, max = 67). Detailed demographic statistics are presented in Appendix A Table A1.

### 2.3. Outcome Measures

The primary outcome variables were depressiveness, anxiety, general distress, perceived stress and hopelessness. Social connectedness and fear of COVID-19 were assessed as secondary outcome variables. In the course of the intervention’s duration, the additional secondary outcomes of relaxation and perceived stress were measured, respectively.

### 2.4. Materials

Perceived Stress Scale 10 (PSS-10 [50]; German adaptation: [51]). The PSS-10 was validated in both clinical and nonclinical samples and achieved good reliability (*r* = 0.88 and *r* = 0.89, respectively). Therefore, it is a robust and reliable measurement of perceived stress [51]. Participants rated the ten items of the self-report scale on a 5-point Likert scale ranging from “0 = never” to “4 = very often”. In accordance with Riva et al. [8], the instructions of the present study were adapted to assess feelings of perceived stress within the last week instead of the last month.

Depression Anxiety Stress Scales (DASS). In the present study, the German short version [52] of the DASS [53] was used. It consists of 21 items equally divided into three subscales measuring anxiety, depression, and perceived stress, respectively. Participants rated how they felt in the previous seven days on a 4-point Likert scale ranging from “0 = did not apply to me at all” to “3 = applied to me very much, or most of the time” [8]. Each subscale can be computed individually or added together into a score for general distress. The internal consistency of the scales is *α* = 0.88 for depression, α = 0.76 for anxiety, and α = 0.86 for stress [54]. Good reliability scores for the depression (0.91), anxiety (0.82), and stress (0.89) subscales were achieved [52].

Social Connectedness Scale (SCS) [55]. The SCS measures whether the individual feels connected to other people and to the social context. The short version consists of eight items rated on a 6-point Likert scale ranging from “6 = strongly disagree” to “1 = strongly agree”. The original short-scale achieved very good reliability scores (*r* = 0.91) [55]. For the current study, the SCS was translated into German using a back-translation technique. The calculated Cronbach’s alpha indicates a good internal consistency (α = 0.93).

Beck Hopelessness Scale (BHS [56]; German adaptation [57]). The BHS measures one’s pessimistic expectations of the future. Consisting of twenty items with a true/false response choice, it captures the three major aspects of hopelessness (feelings concerning the future, expectations, loss of motivation). The reliability of the BHS in a representative German sample was *r* = 0.87 [58].

Fear of COVID-19 scale (FCV-19S) [59]. The FCV-19S captures the level of fear regarding the COVID-19 pandemic. It contains seven items exploring different aspects of fear (i.e., personal experience of concern regarding the current situation, avoidance behaviors, attentional bias) on a 5-point Likert scale ranging from “1 = strongly disagree” to “5 = strongly agree”. A Cronbach’s alpha of α > 0.70 for the different scales was found [59]. For the purpose of this study, the FCV-19S was translated into German, using a back-translation technique. The calculated Cronbach’s alpha indicates a good internal consistency (α = 0.87).

Smith Relaxation State Inventory 3 (SRSI3) [60]. The SRSI3 consists of 38 items, measuring current relaxation and perceived stress (e.g., “How do you feel right now?”) on a 6-point Likert scale ranging from “1 = not at all” to “6 = maximum”. In accordance with Riva et al. [8], only 20 out of the 38 items were selected for the present study, including the following subscales: rest/refresh, energized, physical relaxation, at ease/peace, joy, mental quiet, awareness, somatic stress, emotional stress, and cognitive stress. Internal consistency ranges from α = 0.60 to α = 0.88 [60]. The original version of the SRSI3 was translated into German using a back-translation technique. Cronbach’s alphas were calculated for the utilized subscales, ranging from α = 0.57 to α = 0.83.

Subjective Units of Distress Scale (SUDS) [61]. The SUDS assesses the perceived level of distress rated by the participant on a numeric scale from 0 to 100. Higher scores indicate higher levels of distress.

### 2.5. The 7-Day Self-Help Protocol

In the course of the duration of the 7-day self-help program, a different exercise had to be performed each day (see Table 1). The content of these exercises was inspired by Winch [40], adopted by Riva et al. [8], and aimed at reinforcing coping skills, protecting self-esteem as well as recognizing emotional discomfort, finding personal meaning even in difficult times and eventually revising core assumptions and beliefs [49]. The protocol can be found in the Supplementary Materials.

### 2.6. The Secret Garden Video

The rationale of the 360° VR video *Secret Garden* was based on elements of compassion-focused therapy. It is designed as a digital “safe haven”, a place of relaxation and self-reflection, and was developed in an integrated process involving psychologists, 3D artists, musicians, storytellers, and designers [8]. It allows the viewer to wander through a simulated Asian garden. Furthermore, 360° videos offer a new technological way to make virtual environments tangible by inducing the feeling of immersion and interaction with the environment of the virtual world: viewers can take a “look around” while watching the video and therefore view the *Secret Garden* from different perspectives [62]. To make the VR experience affordable and easily accessible to participants, simple cardboard VR headsets (Basetech Headmount Google 3D), compatible with smartphone displays from 3.5″ (8.9 cm) to 6.0″ (15.2 cm), were utilized. The VR headsets were sent to each participant.

### 2.7. Procedure

The study was conducted between January and May 2021 and approved by the local ethics committee of the Private University of Applied Science in Goettingen, Germany (application number: 251983). Informed consent was obtained from all subjects involved in the study.

The study procedure consisted of a baseline measurement on day −7, a pre-measurement on day 0 (before the start of the program), a post-measurement on day 7 (end of the program), and a follow-up measurement on day 21 (two weeks after the end of the program). On the first day of the waiting list condition (day −7), participants filled in an online questionnaire concerning demographic information and a battery of semi-trait questionnaires (DASS, PSS-10, BHS, SCS, FCV-19S). The same questionnaires were again sent to participants the day before the start of the protocol (day 0). On the last day of the intervention protocol (day 7), participants filled in the same semi-trait questionnaires. Additionally, they were asked to complete the Negative Effect Questionnaire (NEQ) [63], the Simulation Sickness Questionnaire (SSQ) [64], and a final interview to evaluate the feasibility and handling of the self-help protocol. To monitor state relaxation and stress, the state questionnaires (SRSI3 and SUDS) were collected throughout the intervention from day 1 to 7. To assess the stability of potential treatment effects, participants were again asked to fill in the DASS, PSS-10, BHS, SCS and FCV-19S at a 2-week follow-up (day 21).

Participants received the self-help protocol, including instructions and a link to the *Secret Garden* video via e-mail. A head-mounted display was provided if the participants did not own one. For the duration of the intervention (day 1 to day 7), participants followed the same procedure every day. They started the self-help protocol with the daily VR experience of the *Secret Garden*. After having completed the video, participants worked on the respective daily exercise [8]. Every exercise had to be performed by oneself first and was discussed with the partner afterwards. The exercises had to be completed in written form. By combining the VR experience *Secret Garden* with the daily social tasks, the self-help program provides the possibility of immersing oneself in a “safe haven” far from the stressful daily pandemic context, without entirely disconnecting this safe space from the real world. The social tasks bridge the gap to transfer these acquired reflections to prominent real-world problems and solutions [49].

## 3. Results

The statistical analyses were conducted using IBM SPSS Statistics 27. The data was preprocessed using MS Excel 10.

### 3.1. Demographics

Most of the participants (74%) reported being currently employed. More than half of the participants reported their marital status as being single (unmarried) (55%). Five out of thirty-eight participants reported to be suffering from mental disorders, such as depression (*n* = 2), anxiety disorders (*n* = 2) and substance abuse (*n* = 1). Over half of the participants (51%) were registered in the German federal state of Lower Saxony (see Appendix A Table A1 for detailed demographic information).

### 3.2. Hypothesis Testing

All hypotheses were tested with a repeated measure 1 × 4 ANOVA with post hoc pairwise comparisons.

**Figure 1:**
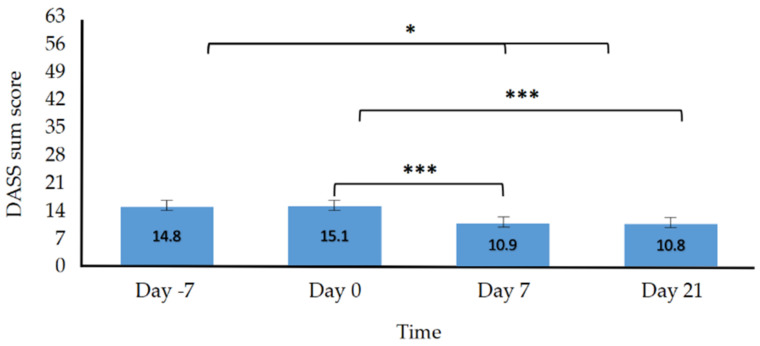
General Distress. Note. Depression Anxiety Stress Scale (DASS), *F*(3, 111) = 11.65, *p* < 0.001, error bars: ±2 SE, average sum score on the y-axis, * *p* < 0.05, *** *p* < 0.001.

**Figure 2:**
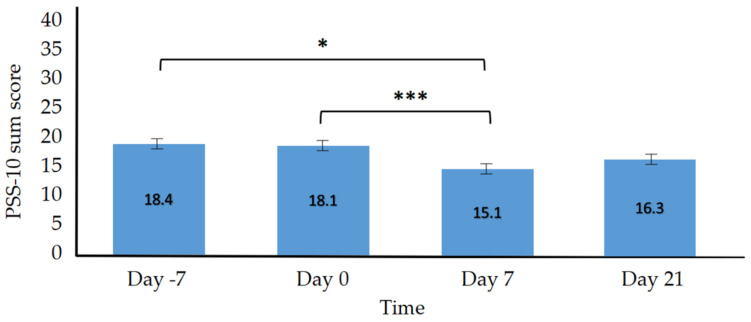
Perceived Stress. Note. Perceived Stress Scale 10 (PSS-10), *F*(3, 111) = 4.74, *p* = 0.004, error bars: ±2 SE, average sum score on the y-axis, * *p* < 0.05, *** *p* < 0.001.

For the primary outcomes, we expected the implementation of the VR self-help program to lead to a significant reduction in depression, anxiety, perceived stress and hopelessness. The scores on the variables described should therefore be significantly lower after the intervention in comparison to before. In line with this prediction, there was a statistically significant effect of the variable *Time* displaying difference between the four measurements (day −7, day 0, day 7, and day 21) for general distress (*F*(3, 111) = 11.65, *p* < 0.001, η^2^ = 0.061; see Figure 1), as well as the subscales depression (*F*(3, 111) = 7.93, *p* = < 0.001, η^2^ = 0.047), anxiety (*F*(3, 111) = 7.80, *p* < 0.001, η^2^ = 0.047), and stress (*F*(3, 111) = 6.78, *p* < 0.001, η^2^ = 0.052). The main effect of *Time* for perceived stress was also statistically significant (*F*(3, 111) = 4.74, *p* = 0.004, η^2^ = 0.038; see Figure 2). However, the decrease in hopelessness turned out to be insignificant (*F*(3, 111) = 2.65, *p* = 0.052, η^2^ = 0.009). The descriptives for the primary outcomes are reported in Table 2. The mean scores of the DASS subscales are depicted in Table 3. The results of the Bonferroni-adjusted post hoc analysis of the primary outcomes can be found in Table 4. In line with the hypothesis, the comparison between day −7 and day 0 indicated no significant changes among all primary outcomes nor for the DASS subscales. Participants showed improvement from day 0 to day 7 for all primary outcomes (*p* < 0.01) and the DASS subscales depression (*p* < 0.01), anxiety (*p* < 0.05) and stress (*p* < 0.001). Only perceived hopelessness turned out to be insignificant (*p* = 0.09). In addition, general distress was significantly lower on day 21 than on day 0 (*p* < 0.001) as were the subscales of depression (*p* < 0.01), anxiety (*p* < 0.01), and stress (*p* = 0.02). A detailed analysis of the DASS subscales can be found in Appendix A Table A2.

For the secondary outcomes, we expected an increase in social connectedness and a decrease in fear of coronavirus as a result of the VR self-help program. Indeed, there was a statistically significant main effect of *Time* for social connectedness (*F*(3, 111) = 5.49, *p* < 0.01, η^2^ = 0.018) and for fear of coronavirus (*F*(3, 111) = 10.92, *p* < 0.001, η^2^ = 0.042). For descriptives, see Table 2 and for the Bonferroni-adjusted post hoc analysis, see Table 4. In accordance with our hypothesis, participants showed improvement in social connectedness from day 0 to day 7 (*p* < 0.01). A significant reduction in the fear of coronavirus was found for days −7 to 0 (*p* = 0.02), but not as hypothesized from day 0 to day 7 (*p* = 0.43). In addition, the reduction in fear of coronavirus was also significantly reduced from day 0 to day 21 (*p* = 0.01).

**Figure 3:**
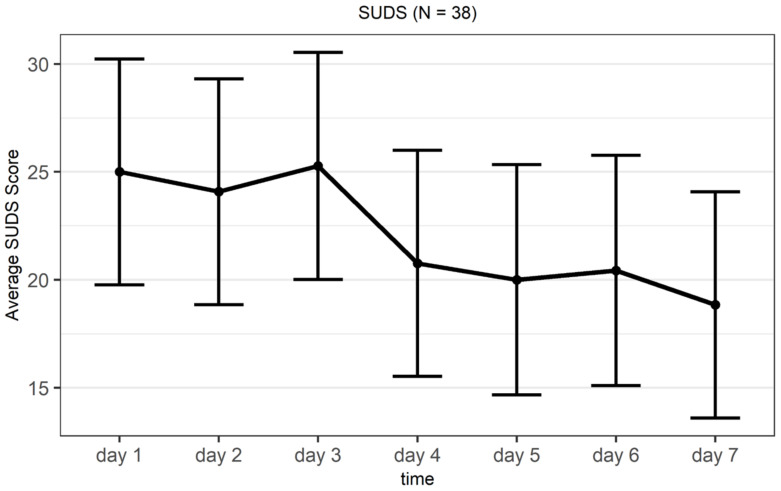
Average Subjective Units of Distress Scale (SUDS) scores and standard errors for the seven trial days.

**Figure 4:**
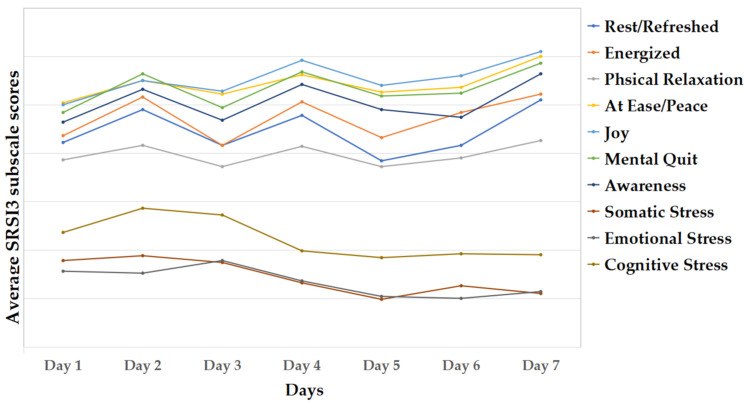
Average scores for the Smith Relaxation States Inventory 3 (SRSI3) subscales.

For the state measures, a reduction in subjective distress measured by the SUDS during the program’s run was expected. In line with this prediction, there was a statistically significant difference in subjective distress (*F*(6, 186) = 3.99, *p* = 0.013, η^2^ = 0.052) comparing the first and the last day of the program (day 1 and day 7). See Figure 3 for the scores of subjective distress displaying all the days of the program. For the SRSI, we expected a significant increase in all subscales except the three stress-related scales, for which a significant reduction was expected. While most of the pairwise comparisons turned out to be non-significant (see Appendix A Table A3), we observed tendencies in the expected direction, as depicted in Figure 4.

## 4. Discussion

The aim of this study was to evaluate the effectiveness of a novel VR self-help protocol from Riva et al. [8] which was designed to help participants cope with the psychological burden associated with the COVID-19 pandemic.

There were no significant changes in the outcome measures comparing the beginning (day −7) to the end (day 0) of the waiting list period except for the fear of coronavirus. In line with hypotheses, the participants experienced significantly lower levels of perceived stress, depressive mood, anxiety, stress, and general distress at the end of the intervention (day 7) in comparison to before the start of the intervention (day 0). The feeling of social connectedness increased significantly after the intervention. Although the average level of hopelessness decreased throughout the course of the program, this decrease was shown to be insignificant. The participants’ feelings of general distress, depression, anxiety, and stress were significantly lower at the 2-week follow-up (day 21) compared to the start of the program (day 0), confirming the stability of treatment effects. While this suggests a long-lasting treatment effect of the program in reducing negative emotions, it could also in part be explained by decreasing COVID-19 case numbers and the easing of restriction measures during the survey period.

In addition, we observed a reduction in subjective distress, as well as an increase in relaxation during the intervention period of the program. Interestingly, the results indicated an abrupt decrease in state distress on day 4 compared to the previous three days of the program, which stayed fairly consistent for the rest of the trial (see Figure 4). The respective exercise of day 4 might have played a key role in that regard as it was aimed at enhancing the sense of community, which directly targets one of the main dilemmas associated with the COVID-19 pandemic [8]. The findings suggest that this exercise was very effective in achieving this goal.

Our results are in line with current literature suggesting that self-guided interventions can help isolated individuals manage their depression, stress, anxiety, and well-being at home during the COVID-19 pandemic (see [65]); especially stress and anxiety levels seem to be lowered by online self-help interventions [66,67]. Further beneficial effects on mental health during the pandemic were achieved by employing videos with nature content [68]. Moreover, [69] showed that VR techniques can help manage the potential short- and long-term psychological consequences of the COVID-19 pandemic, such as stress.

Furthermore, the results from our German sample are largely consistent with the results of Riva et al. [49]. Lower levels of perceived stress (PSS-10) and general distress, depression, and stress (DASS) at the end of the intervention (day 7) in comparison to before the program (day 0) were also reported in the Italian sample. A reduction in anxiety from day 0 to 7 was only found in the German sample. Changes in hopelessness (BHS) turned out to be insignificant in both samples, whereas the feeling of social connectedness (SCS) increased significantly from day 0 to day 7 in both samples. The present study, as well as the study conducted by Riva et al. [49], both found stability of treatment effects for general distress (DASS) comparing day 0 to day 21. Additionally, in our German sample, all subscales of the DASS remained statistically significant at follow-up, whereas in the Italian sample, only the subscale stress remained significant. However, a significant decrease in perceived stress (PSS-10), as well as a significant increase in social connectedness (SCS) from day 0 to day 21 was only found in the Italian sample by Riva et al. [49].

Moreover, according to Riva et al. [49], the modality of the program, i.e., immersive/non-immersive, does not seem to significantly influence the treatment effect. Since VR is not obligatory, the program might therefore prove suitable for a more general population.

### 4.1. Limitations

While the findings of this trial are promising, it should be noted that the program is based on a technology that still has much potential for improvement before the general population can access a high-quality VR experience. While feedback on the protocol during the final interview on day 7 was overall very positive, we received a number of reports concerning the low display quality and resolution, the experience of motion sickness, as well as discomfort while wearing the cardboard glasses, which led to a distraction from the relaxation process. Further technological and application improvements are required to make cost-efficient, yet high-quality VR headsets more widely available, thus enabling VR-based interventions to reach their full potential to improve mental health.

An extensive limitation of the present study is the lack of a separate control group and therefore missing randomization. It remains uncertain if the effects found can be attributed solely to the specific tasks of the self-help protocol. It can thus be argued that simply doing any kind of task on a daily basis may prove beneficial in reducing psychological burden simply by having an activating effect and by structuring one’s time during nationwide restrictions. To rule out this possible demand effect [70] in future studies, it is necessary to use a randomized control trial comparing the program with an active control group that is participating in alternative tasks of a similar length, not explicitly designed to reduce the psychological burden. Nonetheless, given the specific context of the intervention, the primary aim of this multicentric pragmatic pilot trial was to provide each participant with the opportunity to benefit from the use of the protocol. Therefore, a waiting list design was used instead of an RCT design.

Furthermore, the sample is also susceptible to criticism. After all, it is a convenience sample with limited representativeness, making it difficult to draw general conclusions about the population. Only participants having access to an internet-enabled smartphone were considered. Furthermore, two-thirds of the participants were female. This gender imbalance may have had an impact on the results, as females generally showed a higher degree of distress due to the pandemic [17,46]. The majority of this sample consisted of nonclinical participants, though depression and stress scores before the start of the intervention were comparatively high for a convenience sample. However, our sample included five participants who reported having been previously diagnosed with a mental disorder. Nevertheless, reported measures did not seem to be significantly influenced by the diagnoses as results remained statistically significant even after the exclusion of these participants; even though p values slightly increased in some measures. Effects sizes slightly decreased after removing these participants, which is in line with empiric evidence that effect sizes in convenience samples tend to be smaller in comparison to clinical samples [71]. Therefore, our program may lead to larger treatment effects in a population diagnosed with a mental illness.

Aside from that, the sample represents only 9 out of 16 German federal states, with half of the sample (51.35%) being citizens of Lower Saxony (see Appendix A Table A1 for sample distribution by federal state). No participants from the eastern German federal states were represented in this study, though the COVID-19 infection rates were especially high in those states [72]. This may affect the generalizability of our results, as regional differences in governmental restrictions and measures seem to have led to differences in coping with strains during the pandemic [73,74].

Moreover, the time of the survey may have led to an overestimation of reduction regarding the fear of coronavirus (FCV-19S). During January and May 2021, vaccination rates in Germany increased while the number of confirmed cases decreased in many federal states [72]. Furthermore, our program was not specifically aimed at reducing the fear of coronavirus but rather at reducing general anxiety and stress; therefore, a higher impact of environmental circumstances seems reasonable.

In addition, the state measures of relaxation and perceived distress (SRSI3 & SUD) were only monitored during the intervention phase (day 1 to day 7) but not assessed during the waiting list period (day −7 to day 0); future studies should do so. Further, whether participants continued to perform the exercises of the self-help protocol even after the end of the intervention during the two-week follow-up was not assessed; this might account for the stability of treatment effects regarding general distress.

Finally, it must be mentioned that the tools SCS, FCV-19S, and SRSI3 have been translated from English into German, with no validation of quality criteria. Though a back-translation technique was used to lower the impact of translation and calculated Cronbach’s alphas indicates a good internal consistency, it cannot be completely ruled out that the independent translation could have influenced the response behavior of the participants.

### 4.2. Implications

Self-help programs like the *Secret Garden* offer the immense advantage that realistic natural environments can be experienced in times of distress, such as the ongoing pandemic, and could be applied in other situations where the possibility for personal movement is limited (e.g., during the winter months or for people in large cities with limited access to nature) and where the susceptibility for psychological burden is higher [75,76,77].

Furthermore, future self-help interventions should focus on the needs of vulnerable groups that are particularly burdened by the COVID-19 pandemic, such as women [7], young people [14,16,78], and people with chronic illnesses [16] and mental disorders [43]. Clearly, easier-to-use and scientifically evaluated self-help programs that are free of charge and accessible to everyone are needed in the near future to provide tailored solutions for different (at risk) groups and to boost individuals’ resilience. They could help to bridge the gap between the lack of available public health services and the increased number of individuals seeking help from health care professionals [79]. As digital and self-help options provide more choices and solutions and allow patients to make progress, even without active guidance from a therapist, these alternatives may reduce the supply gap, especially for those living in rural areas [80].

## 5. Conclusions

Events such as the COVID-19 pandemic have a significant impact on the economy and society, but the psychological consequences may also be long-lasting. To reduce harmful long-term consequences, there is a need for evidence-based interventions to cope with the psychological challenges related to the COVID-19 pandemic. The present study pointed out that VR-based self-help protocols can mitigate the stress associated with the pandemic. The German version of the *COVID Feel Good* self-help program seems to be an effective tool in reducing negative feelings, supporting the findings of Riva et al. [49]. Although the study has some limitations and the results were not significant for every outcome, the findings were overall satisfying and can serve as an indication of the effectiveness of this program. Further studies are necessary to explore the effectiveness of the protocol in other groups, countries, or contexts.

## Figures and Tables

**Table 1:** Description of daily exercises.

Day 1: ***Fight rumination***. Support to cope with stress, worries, and negative intrusive thoughts related to the COVID-19 pandemic through imagination exercises.
Day 2: ***Awaken self-esteem***. Increase self-esteem by listing five aspects of one’s own character and personality that one appreciates.
Day 3: ***Awaken autobiographical memory***. Aims at creating a stable representation of themselves over time, as well as increase intimacy and connectedness by sharing personal memories.
Day 4: ***Awaken sense of community***. Aims at reducing the feeling of loneliness by focusing on the five most important people in one’s life.
Day 5: ***Awaken goals and dreams***. Promote conscious self-regulation and self-organization of life goals by listing concrete goals, dreams, and aspirations.
Day 6: ***Boost empathy***. Increase in empathy by attributing feelings to last significant interactions with the most significant people in one’s life.
Day 7: ***Plan change***. Support a long-term psychological change by finding solutions for life dissatisfactions.

Note. The social tasks relate to interpersonal relationships and personal identity. For details, see [8].

**Table 2:** Descriptive Statistics for Outcome Measures.

Primary Outcome Measures	Day −7 Mean (SD)	Day 0 Mean (SD)	Day 7 Mean (SD)	Day 21 Mean (SD)
DASS	14.79 (8.44)	15.13 (8.87)	10.92 (8.01)	10.79 (7.34)
PSS-10	18.39 (6.62)	18.08 (6.83)	15.11 (7.20)	16.26 (6.92)
BHS	4.84 (3.90)	4.68 (4.27)	3.92 (3.92)	4.13 (4.32)
**Secondary Outcome Measures**	**Day −7** **Mean (SD)**	**Day 0** **Mean (SD)**	**Day 7** **Mean (SD)**	**Day 21** **Mean (SD)**
SCS	35.84 (7.90)	36.34 (9.26)	38.79 (8.97)	38.00 (9.38)
FCV-19S	14.47 (4.30)	13.34 (4.39)	12.66 (5.27)	11.87 (4.52)

Note. Descriptives for the primary and secondary outcome variables by time points (day −7 = baseline; day 0 = before start of intervention; day 7 = end of intervention; day 21 = 2-week follow-up). Data are provided in means and standard deviations (SD) in parentheses. Depression Anxiety Stress Scale: DASS; Perceived Stress Scale: PSS-10; Beck Hopelessness Scale: BHS; Social Connectedness Scale: SCS; Fear of COVID-19 scale: FCV-19S.

**Table 3:** Descriptive Statistics for Depressive Anxiety Stress Scale subscales.

Subscales	Day −7 Mean (SD)	Day 0 Mean (SD)	Day 7 Mean (SD)	Day 21 Mean (SD)
Depression	9.16 (7.32)	9.42 (7.23)	6.68 (6.63)	6.05 (5.72)
Anxiety	4.95 (4.89)	4.95 (5.34)	3.47 (3.80)	2.74 (2.99)
Stress	15.47 (7.82)	15.89 (7.89)	11.68 (7.17)	12.79 (7.61)

Note. Descriptives for the Depressive Anxiety Stress Scale (DASS) subscales (depression, anxiety, and stress) by time points (day −7 = baseline; day 0 = before start of intervention; day 7 = end of intervention; day 21 = 2-week follow-up). Data are provided in means and standard deviations (SD) in parentheses.

**Table 4:** Bonferroni-adjusted Pairwise Comparisons for Outcome Measures.

	Contrasts		Estimate	*p*-Value	Lower Limit	Upper Limit
DASS	Day −7	Day 0	−0.34	1.00	−1.70	Inf
		Day 7	3.87 *	0.05	2.07	Inf
		Day 21	4.00 *	0.04	2.19	Inf
	Day 0	Day 7	4.21 *	0.00	2.61	Inf
		Day 21	4.34 *	0.00	2.81	Inf
	Day 7	Day 21	0.13	1.00	−1.68	Inf
PSS-10	Day −7	Day 0	0.34	1.00	−0.98	Inf
		Day 7	3.29 *	0.02	1.34	Inf
		Day 21	2.13	0.22	0.20	Inf
	Day 0	Day 7	2.97 *	0.00	1.54	Inf
		Day 21	1.82	0.22	0.17	Inf
	Day 7	Day 21	−1.16	1.00	−2.99	Inf
BHS	Day −7	Day 0	0.16	1.00	−0.27	Inf
		Day 7	0.92	0.06	0.29	Inf
		Day 21	0.71	0.38	−0.06	Inf
	Day 0	Day 7	0.76	0.09	−0.19	Inf
		Day 21	0.55	0.50	−0.22	Inf
	Day 7	Day 21	−0.21	1.00	−0.83	Inf
SCS	Day −7	Day 0	−0.50	1.00	−Inf	0.79
		Day 7	−2.95 *	0.01	−Inf	−1.49
		Day 21	−2.16	0.16	−Inf	−0.32
	Day 0	Day 7	−2.45 *	0.00	−Inf	−1.44
		Day 21	−1.66	0.16	−Inf	−0.24
	Day 7	Day 21	0.79	1.00	−Inf	2.10
FCV-19S	Day −7	Day 0	1.13 *	0.02	0.50	Inf
		Day 7	1.82 *	0.01	0.91	Inf
		Day 21	2.61 *	0.00	1.74	Inf
	Day 0	Day 7	0.68	0.43	−0.08	Inf
		Day 21	1.47 *	0.01	0.67	Inf
	Day 7	Day 21	0.79	0.30	0.01	Inf

Note. Bonferroni-adjusted pairwise comparisons for all primary and secondary outcome measures (semi-trait measures) across different time points (day −7 = baseline; day 0 = before start of intervention; day 7 = end of intervention; day 21 = 2 week-follow-up). * *p* < 0.05. 95%—Confidence Interval. Depression Anxiety Stress Scale: DASS; Perceived Stress Scale: PSS-10; Beck Hopelessness Scale: BHS; Social Connectedness Scale: SCS; Fear of COVID-19 scale: FCV-19S.

## Data Availability

The data presented in this study are available on request from the corresponding author. The data are not publicly available due to this study being part of an ongoing project. Data will be made publicly available once the overall project is completed.

## Supplementary Materials

The following supporting information can be downloaded at: https://www.covidfeelgood.com/das-selbsthilfe-verfahren-deutsche-version (accessed on 30 January 2021). Video: Augmented Relaxation—der Geheime Garten. Protocol: Das Selbsthilfe-Verfahren (Deutsche Version).

## Appendix A

**Table A1:** Demographics.

Marital Status	*N* (Male)	*N* (Female)	*N* (Total)
Divorced	0 (0.00%)	3 (10.71%)	3 (7.89%)
Married	6 (60.00%)	7 (25.00%)	13 (34.21%)
Separated	0 (0.00%)	1 (3.57%)	1 (2.63%)
Single	4 (40.00%)	17 (60.71%)	21 (55.26%)
Total	10 (100%)	28 (100%)	38 (100%)
Employment Status			
Office employee	1 (10.00%)	4 (14.29%)	5 (13.16%)
Pensioners	1 (10.00%)	2 (7.14%)	3 (7.89%)
Student	0 (0.00%)	6 (21.43%)	6 (15.79%)
Unemployed	0 (0.00%)	1 (3.54%)	1 (2.63%)
Worker	8 (80.00%)	15 (53.57%)	23 (60.53%)
Total	10 (100%)	28 (100%)	38 (100%)
State			
Berlin	0 (0.00%)	3 (11.11%)	3 (8.11%)
Baden-Wuerttemberg	0 (0.00%)	0 (0.00%)	2 (5.41%)
Bavaria	2 (20.00%)	1 (3.78%)	1 (2.70%)
Hesse	0 (0.00%)	1 (3.78%)	2 (5.41%)
Hamburg	1 (10.00%)	2 (7.44%)	4 (10.81%)
Lower Saxony	2 (20.00%)	15 (50.56%)	19 (51.35%)
North Rhine Westphalia	4 (40.00%)	1 (3.78%)	1 (2.70%)
Rhineland-Palatinate	0 (0.00%)	0 (0.00%)	1 (2.70%)
Schleswig-Holstein	1 (10.00%)	4 (14.81%)	4 (10.81%)
Total	10 (100%)	27 (100%)	37 (100%)
Known Disorder			
No	10 (100%)	23 (82.14%)	33 (86.84%)
Yes	0 (0.00%)	5 (17.86%)	5 (13.16%)
Total	10 (100%)	28 (100%)	38 (100%)
Type of Disorder			
Depression	0 (0.00%)	2 (100%)	2 (5.26%)
Anxiety	0 (0.00%)	2 (100%)	2 (5.26%)
Substance Addiction	0 (0.00%)	1 (100%)	1 (2.63%)

Note. Only 9 out of 16 German federal states are represented. The known disorders were depression, anxiety and obsessive-compulsive disorder, addiction disorder, and exam anxiety. The participant with the addiction disorder was reported to be under treatment.

**Table A2:** Bonferroni-adjusted Pairwise Comparisons for Depression Anxiety Stress Scale subscales.

	Contrasts		Estimate	*p*-Value	Lower Limit	Upper Limit
Depression	Day −7	Day 0	−0.26	1.00	−2.35	Inf
		Day 7	2.47 *	0.03	0.02	Inf
		Day 21	3.11 *	0.00	0.63	Inf
	Day 0	Day 7	2.73 *	0.01	0.35	Inf
		Day 21	3.69 *	0.00	0.99	Inf
	Day 7	Day 21	0.63	1.00	−1.82	Inf
Anxiety	Day −7	Day 0	0.00	1.00	−1.38	Inf
		Day 7	1.47	0.08	−0.29	Inf
		Day 21	2.21 *	0.00	0.61	Inf
	Day 0	Day 7	1.47	0.04	−0.12	Inf
		Day 21	2.21 *	0.00	0.61	Inf
	Day 7	Day 21	0.74	0.42	−0.62	Inf
Stress	Day −7	Day 0	−0.42	1.00	−3.01	Inf
		Day 7	3.79 *	0.01	0.63	Inf
		Day 21	2.68	0.13	−0.91	Inf
	Day 0	Day 7	4.21 *	0.00	1.32	Inf
		Day 21	3.11 *	0.02	0.17	Inf
	Day 7	Day 21	−1.11	1.00	−4.45	Inf

Note. Bonferroni-adjusted pairwise comparisons for all Depression Anxiety Stress Scale (DASS) subscales (depression, anxiety, stress) across different time points (day −7 = baseline; day 0 = before start of intervention; day 7 = end of intervention; day 21 = 2-week follow-up. * *p* < 0.05. 95%—Confidence Interval.

**Table A3:** Pairwise Comparisons for Smith Relaxation States Inventory 3 subscales.

	Contrasts		Estimate	*p*-Value	Lower Limit	Upper Limit
Rest/refresh	Day 1	Day 7	−0.34	0.078	−Inf	0.06
Energized	Day 1	Day 7	−0.39	0.027 *	−Inf	−0.06
Physical Relaxation	Day 1	Day 7	−0.14	0.197	−Inf	0.14
At Ease/Peace	Day 1	Day 7	−0.24	0.059	−Inf	0.01
Joy	Day 1	Day 7	−0.25	0.066	−Inf	0.02
Mental Quiet	Day 1	Day 7	−0.39	0.014 *	−Inf	−0.1
Awareness	Day 1	Day 7	−0.34	0.057	−Inf	0.01
Somatic Stress	Day 1	Day 7	−0.05	0.661	−0.27	Inf
Emotional Stress	Day 1	Day 7	0.02	0.445	−0.24	Inf
Cognitive Stress	Day 1	Day 7	−0.25	0.93	−0.54	Inf

Note. Bonferroni-adjusted pairwise comparisons for all Smith Relaxation States Inventory 3 (SRSI 3) subscales comparing the first (day 1) and the last day (day 7) of the 7-day self-help program *COVID Feel Good*. * *p* < 0.05. 95%—Confidence Interval.
